# Hypertensive *APOL1* risk allele carriers demonstrate greater blood pressure reduction with angiotensin receptor blockade compared to low risk carriers

**DOI:** 10.1371/journal.pone.0221957

**Published:** 2019-09-18

**Authors:** Patrick N. Cunningham, Zhiying Wang, Megan L. Grove, Rhonda M. Cooper-DeHoff, Amber L. Beitelshees, Yan Gong, John G. Gums, Julie A. Johnson, Stephen T. Turner, Eric Boerwinkle, Arlene B. Chapman

**Affiliations:** 1 Section of Nephrology, University of Chicago, Chicago, Illinois, United States of America; 2 Human Genetics Center, Department of Epidemiology, Human Genetics and Environmental Sciences, School of Public Health, The University of Texas Health Science Center at Houston, Houston, Texas, United States of America; 3 Department of Pharmacotherapy and Translational Research, College of Pharmacy and Division of Cardiovascular Medicine, College of Medicine, University of Florida, Gainesville, Florida, United States of America; 4 Endocrinology, Diabetes, and Nutrition Division, Department of Medicine, University of Maryland, Baltimore, Maryland, United States of America; 5 Division of Nephrology and Hypertension, Mayo Clinic, Rochester, Minnesota, United States of America; 6 Baylor College of Medicine, Human Genome Sequencing Center, Houston, Texas, United States of America; International University of Health and Welfare, School of Medicine, JAPAN

## Abstract

**Background:**

Hypertension (HTN) disproportionately affects African Americans (AAs), who respond better to thiazide diuretics than other antihypertensives. Variants of the *APOL1* gene found in AAs are associated with a higher rate of kidney disease and play a complex role in cardiovascular disease.

**Methods:**

AA subjects from four HTN trials (n = 961) (GERA1, GERA2, PEAR1, and PEAR2) were evaluated for blood pressure (BP) response based on *APOL1* genotype after 4–9 weeks of monotherapy with thiazides, beta blockers, or candesartan. *APOL1* G1 and G2 variants were determined by direct sequencing or imputation.

**Results:**

Baseline systolic BP (SBP) and diastolic BP (DBP) levels did not differ based on *APOL1* genotype. Subjects with 1–2 *APOL1* risk alleles had a greater SBP response to candesartan (-12.2 +/- 1.2 vs -7.5 +/- 1.8 mmHg, *p* = 0.03; GERA2), and a greater decline in albuminuria with candesartan (-8.3 +/- 3.1 vs +3.7 +/- 4.3 mg/day, *p* = 0.02). *APOL1* genotype did not associate with BP response to thiazides or beta blockers. GWAS was performed to determine associations with BP response to candesartan depending on *APOL1* genotype. While no SNPs reached genome wide significance, SNP rs10113352, intronic in *CSMD1*, predicted greater office SBP response to candesartan (*p* = 3.7 x 10^−7^) in those with 1–2 risk alleles, while SNP rs286856, intronic in *DPP6*, predicted greater office SBP response (*p* = 3.2 x 10^−7^) in those with 0 risk alleles.

**Conclusions:**

Hypertensive AAs without overt kidney disease who carry 1 or more *APOL1* risk variants have a greater BP and albuminuria reduction in response to candesartan therapy. BP response to thiazides or beta blockers did not differ by *APOL1* genotype. Future studies confirming this initial finding in an independent cohort are required.

## Introduction

Essential hypertension is common and associates with morbidity and mortality with greater rates of cardiovascular, kidney, and cerebrovascular disease. Epidemiologic studies show that essential hypertension (HTN) is more common in AAs and that they have higher rates of cardiovascular and renal failure in comparison to non-African populations [1].

The association between two variant alleles in the *APOL1* gene found primarily in individuals of sub-Saharan ancestry and a number of kidney diseases has been reported [2–4]. However, not all individuals with two risk alleles develop diagnosed kidney disease, and less is known about the natural history of those with a single *APOL1* risk allele. Risk variants in *APOL1* are reported to be associated with increased cardiovascular disease, even without kidney disease [5, 6]. However, other prospective cohorts report fewer cardiovascular events, less vascular calcification and less cerebral microvascular disease in those with two *APOL1* risk alleles [7–9]. In addition, AA hemodialysis patients with two risk alleles have lower mortality rates than controls [10]. The mechanisms by which the *APOL1* gene contributes to renal disease risk and potential cardiovascular outcomes are questions that currently remain largely unanswered. Recent work has examined the effect of *APOL1* genotype on BP in young AAs without diagnosed kidney disease, but has yielded mixed results [11–13].

Studies of HTN patients demonstrate significant differences between Caucasians and AAs [14, 15]. AA HTN patients have lower circulating renin and aldosterone levels, are more salt sensitive, demonstrate less blood pressure response to inhibitors of the renin-angiotensin system, and greater blood pressure response to diuretics [16, 17]. The GERA1 (Genetic Epidemiology of Responses to Antihypertensives) and PEAR1 (Pharmacogenomic Evaluation of Antihypertensive Responses) studies, and the subsequent clinical trials GERA2 and PEAR2, examined epidemiologic and genetic predictors of antihypertensive response in essential hypertensives without kidney disease, in response to a variety of antihypertensive monotherapies. GERA1, PEAR1 and PEAR2 evaluated thiazide diuretics (hydrochlorothiazide and chlorthalidone), while PEAR1 and PEAR2 also evaluated beta blocker monotherapy (atenolol and metoprolol) and GERA2 evaluated an angiotensin receptor blocking agent (candesartan). Genome wide association studies (GWAS) done on the Caucasian subset identified candidate SNPs that associated with better BP response to thiazide diuretics [15], and similarly, analysis of the AA subset identified novel SNPs associated with better BP response to beta blockers [18]. These clinical trials with well phenotyped and genotyped essential hypertensive subjects present an opportunity to gain insights and an understanding of the natural history of *APOL1* risk allele carriers in AA hypertensives without evidence of overt kidney disease. In this study we examine differences in baseline characteristics and the response to different antihypertensive agents when administered as monotherapy in this group according to *APOL1* genotype.

## Methods

### Study populations and interventions

Data from this analysis combine patients from four previously completed clinical trials, PEAR1, PEAR2, GERA1, and GERA2, which investigated clinical and genetic predictors of different antihypertensive drug responses in essential hypertensive individuals. All subjects gave written informed consent to participate and supply genetic material. All studies and data analysis were performed in full accordance with Institutional Review Board approval at their respective sites.

Details of these studies have been described previously (www.clinicaltrials.gov, PEAR1: NCT00246519; PEAR2: NCT01203852; GERA1 and GERA2: NCT00005520) [16, 19–21]. In brief, these multicenter trials enrolled patients self-reported as AA or Caucasian who had newly diagnosed, untreated, or known essential hypertension. Patients were recruited at one of three sites (Emory University, Atlanta, GA; Mayo Clinic, Rochester, MN; and University of Florida, Gainesville, Fl). Age ranges varied slightly between studies (age 17–65 in PEAR1 and PEAR2, to age 18–60 in GERA1 and GERA2) but inclusion and exclusion criteria were otherwise similar. After a washout period off all blood pressure medications for at least two weeks, patients with diastolic blood pressure (DBP) > 85 mm Hg (home measurement) and > 90 mm Hg (office measurement) were enrolled. Patients with known heart disease, diabetes, or kidney disease (serum creatinine above 1.5 mg/dl for males or 1.4 mg/dl for females, or proteinuria over 1 gm per day) were excluded. After enrollment, patients were randomized to either hydrochlorothiazide 25 mg or atenolol 100 mg, with one dose titration step followed by assessment six weeks later (PEAR1); metoprolol 100 mg and chlorthalidone 25 mg in a sequential monotherapy design (PEAR2), hydrochlorothiazide 25 mg/day for four weeks (GERA1), or candesartan 16 mg/day for two weeks followed by 32 mg/day for four additional weeks (GERA2). The designs of these trials are summarized in Table 1.

**Table 1:** Combined studies used in this analysis.

Study	Drug intervention	Notes	BP endpoint measured	BP measurement modality	No. AA patients	References
GERA1	Hydrochlorothiazide	Monotherapy, single dose	4 weeks	Clinic n = 280 Ambulatory n = 112	280	16
GERA2	Candesartan	Monotherapy, dose titration	6 weeks	Clinic n = 193	193	19
PEAR	Randomized to hydrochlorothiazide or atenolol	Dose titration after 3 weeks if above BP goal, then addition of other drug if above goal	9 weeks	Clinic n = 298, Home n = 298 Ambulatory n = 253	298	17
PEAR2	Metoprolol → washout for 4 weeks → chlorthalidone	Dose titration of each drug after 2 weeks if above BP goal	8 weeks	Clinic n = 190 Home n = 190	190	18
Total				Clinic, n = 961 Home n = 488 Ambulatory n = 365	961	

### Data collection

This analysis focused exclusively on the subset of patients who were self-identified as AA. Demographic information and baseline clinical variables were collected at enrollment, before introduction of the initial antihypertensive agent. Estimated glomerular filtration rate (eGFR) was calculated using the CKD-EPI equation. Blood pressure at enrollment and after each phase of therapy was measured via home cuff and validated with clinic measurement using that same cuff (PEAR1, PEAR2, and GERA1). PEAR1 and GERA1 studies also recorded 24 hour ambulatory automated measurements taken every 15 minutes during waking hours and every 30 minutes during sleeping hours. Responses to study medications were defined as the difference between blood pressure at baseline and at the end of treatment, and were examined as the primary endpoints.

### Genotyping and quality control

DNA was isolated from blood specimens were taken at enrollment. Various methods were used to determine the genetic variation in *APOL1* rs60910145, rs73885319, and rs71785313 and a summary of data acquisition method, minor allele frequency, SNP call rate, and Rsq is provided in S1 Table.

In brief, rs60910145 and rs73885319 were genotyped in GERA1, GERA2, PEAR1 and PEAR2 using the Illumina HumanExome BeadChip (Illumina, Inc.; San Diego, CA, USA). Genotypes were called using Illumina GenomeStudio software using laboratory best practices described previously by Grove et al. [22]. SNP call rates were > 99% for both SNPs in all four studies. Quality control analyses were performed using PLINK [23]. Samples were excluded if estimated sex using X chromosome markers mismatched reported gender, sample call rate < 97%, identity by descent (IBD) pi-hat > 0.9 (using pruned SNP set which removed variants if missing data > 5%, r^2^ ≥ 0.3, and minor allele frequency [MAF] ≤ 0.05), principal component ± 6 standard deviations (SD) from the mean using same pruned SNP set described previously, and inbreeding coefficient ± 6 SD from the mean. SNPs were excluded if monomorphic, missing data rate > 95%, and Hardy Weinberg Equilibrium *p* < 1 x 10^−6^ if MAF ≥ 0.05 using a chi-square test with one degree of freedom.

Because rs71785313 was not included by the manufacturer in the original microarrays, genotype imputations for rs71785313 were performed using the MaCH software program (version 1.0.16) [24] and 1,000 genome phase 1 reference panel [25]. Detailed imputation methods for PEAR1 and PEAR2 were published previously [18], and GERA2 data was imputed in an analogous fashion. Imputation quality for rs71785313 in PEAR1, PEAR2, and GERA2 were Rsq > 0.90. Imputation quality for rs71785313 was Rsq < 0.9 for GERA1, thus for the GERA1 cohort we performed allelic discrimination using the TaqMan Custom SNP Genotyping Assay (ThermoFisher Scientific; Waltham, MA, USA) in accordance with the manufacturer’s directions. Genotype calling was performed using the ABI 7900HT and the Sequence Detection System software (Applied Biosystems; Foster City, CA, USA).

### Statistics

Genotypes for rs60910145 (A→G) and rs73885319 (T→A) represent the G1 risk allele, and rs71785313 (insertion/deletion) represents the G2 risk allele. These were used to generate genotype s as previously detailed by Papeta et al. [26]. *APOL1* genotype allele frequencies by study are shown in Table 2 with the risk groups coded as 0, 1, or 2. Data were compared between two groups based on both a recessive (0–1 risk allele versus 2 risk alleles) as well as dominant (0 versus 1–2 risk alleles) gene effect, by two sided t test, with *p* < 0.05 considered the threshold for statistical significance. In addition to baseline data, these groups in all studies were compared for change in SBP and DBP in response to antihypertensive monotherapy. This was done for both raw and adjusted BP response; BP response was adjusted for previously identified predictive factors, namely baseline BP, age, gender, principal component 1 (PC 1) and PC 2 [16]. Since only AA participants were analyzed in this study, principal components were derived from this AA dataset only.

**Table 2:** *APOL1* genotype frequencies by study.

	PEAR1		PEAR2		GERA1		GERA2		Risk Alleles
Genotype	N	%	N	%	N	%	N	%	
G0/G0	134	44.97	79	41.58	110	39.29	65	33.68	0
G1/G0	78	26.17	49	25.79	86	30.71	50	25.91	1
G2/G0	50	16.78	30	15.79	47	16.79	41	21.24	1
G1/G1	13	4.36	9	4.74	9	3.21	15	7.77	2
G1/G2	15	5.03	13	6.84	20	7.14	17	8.81	2
G2/G2	8	2.68	10	5.26	8	2.86	5	2.59	2
**Total**	**298**		**190**		**280**		**193**		

### GWAS

Because patients with 1–2 APOL1 risk alleles were found to have a greater SBP response to candesartan versus those with 0 risk alleles, we sought to identify other potential gene variants which could interact with this effect by interaction with *APOL1* genotype. We performed two separate genome wide association studies (GWAS) for each of these two risk groups, looking for associations with adjusted SBP response as the outcome of interest. For this GWAS analysis, a *p* value < 1 x 10^−8^ for each SNP was considered significant.

## Results

Combination of the PEAR1, PEAR2, GERA1, and GERA2 study subjects yielded a total of 961 AA participants, of whom 142 (14.8%) carried two risk alleles for *APOL1*. The breakdown of genotypes in the four component studies are given in Table 2. The baseline characteristics of AA hypertensives with 0–1 versus 2 *APOL1* risk alleles are shown in S2 Table. No significant differences in gender or age were observed, nor were there significant differences in baseline clinic, home, or ambulatory day or night BP, pulse pressure or heart rate. Significant differences in baseline eGFR were found between those with 2 *APOL1* risk alleles versus those with 0–1 risk alleles (98.7 +/- 19.6 versus 104.4 +/- 18.7 ml/min *p* < 0.001), and serum creatinine concentrations were significantly greater in those with two *APOL1* risk alleles (0.93+/- 0.24 versus 0.87+/- 0.21 mg/dl, p = 0.006). Baseline urinary albumin to creatinine ratios did not differ based on *APOL1* genotype (19 +/- 56 versus 30 +/- 90 mcg/mg creatinine in those with 0–1 versus 2 risk alleles, *p* = NS).

Baseline characteristics in AA hypertensives with 0 versus 1–2 *APOL1* risk alleles, consistent with a potential dominant pattern of influence, was also performed, given that it is unclear yet if one or two risk alleles are required to identify an early, relatively asymptomatic phenotype [13, 27]. Those with 1–2 *APOL1* risk alleles had a significantly longer duration of hypertension versus others (8.0 +/- 7.4 versus 6.7 +/- 7.5 years, *p* = 0.02), as well as a borderline significantly higher BMI (31.5 +/- 5.7 versus 30.8 +/- 5.9, *p* = 0.05). No other baseline parameters, including in renal function, were different in those with 0 versus 1–2 risk alleles (shown in Table 3).

**Table 3:** Baseline characteristics.

	APOL1: 0 risk alleles 388 total		APOL1: 1–2 risk alleles 573 total		
	N	Mean (SD)	N	Mean (SD)	P value
Gender (% female)		53.4		57.9	NS
Age	388	47.9 (8.1)	573	48.6 (7.5)	NS
Waist/Hip	307	0.88 (0.08)	456	0.88 (0.08)	NS
BMI	387	30.8 (5.9)	573	31.5 (5.7)	0.05
Hypertension duration	355	6.7 (7.5)	529	8.0 (4.4)	0.01
Hypertension onset age	238	40.2 (9.7)	329	40.1 (9.0)	NS
Albumin (g/dl)	279	3.98 (0.36)	438	3.94 (0.35)	NS
Hemoglobin (g/dl)	309	13.74 (2.18)	462	14.08 (9.39)	NS
Clinic SBP, baseline	388	150.1 (13.8)	573	149.9 (13.4)	NS
Clinic DBP, baseline	388	97.9 (5.9)	573	97.5 (5.9)	NS
Home SBP, baseline	213	146.7 (10.9)	275	146.7 (11.3)	NS
Home DBP, baseline	213	95.0 (6.4)	275	94.9 (6.4)	NS
Ambulatory SBP, all	158	139.8 (11.4)	207	140.3 (12.6)	NS
Ambulatory DBP, all	158	89.0 (8.5)	207	88.7 (8.1)	NS
Ambulatory SBP, day	158	142.3 (11.7)	207	143.0 (12.9)	NS
Ambulatory DBP, day	158	91.4 (8.7)	207	91.3 (8.2)	NS
Ambulatory SBP, night	156	132.6 (12.7)	205	133.9 (14.5)	NS
Ambulatory DBP, night	156	82.2 (9.7)	205	82.4 (10.2)	NS
Urine Na, baseline (meq/24 h)	304	144.9 (78.4)	455	145.5 (65.1)	NS
Serum Na, baseline	386	139.3 (4.4)	571	139.7 (4.6)	NS
Serum K, baseline	385	4.01 (0.45)	569	3.97 (0.39)	NS
Serum creatinine, baseline	386	0.88 (0.22)	572	0.88 (0.22)	NS
Serum aldosterone	371	7.82 (5.94)	558	7.44 (5.43)	NS
Serum renin	385	0.74 (1.48)	571	0.61 (0.65)	NS
Urine alb/creat (mcg/mg)	256	20 (57)	377	25 (100)	NS
eGFR (ml/min/1.73m^2^)	386	104.30 (18.6)	572	103.08 (19.2)	NS

The numbers of patients for different outcome variables differs because not every variable was measured in each of the four component studies.

**Fig 1:**
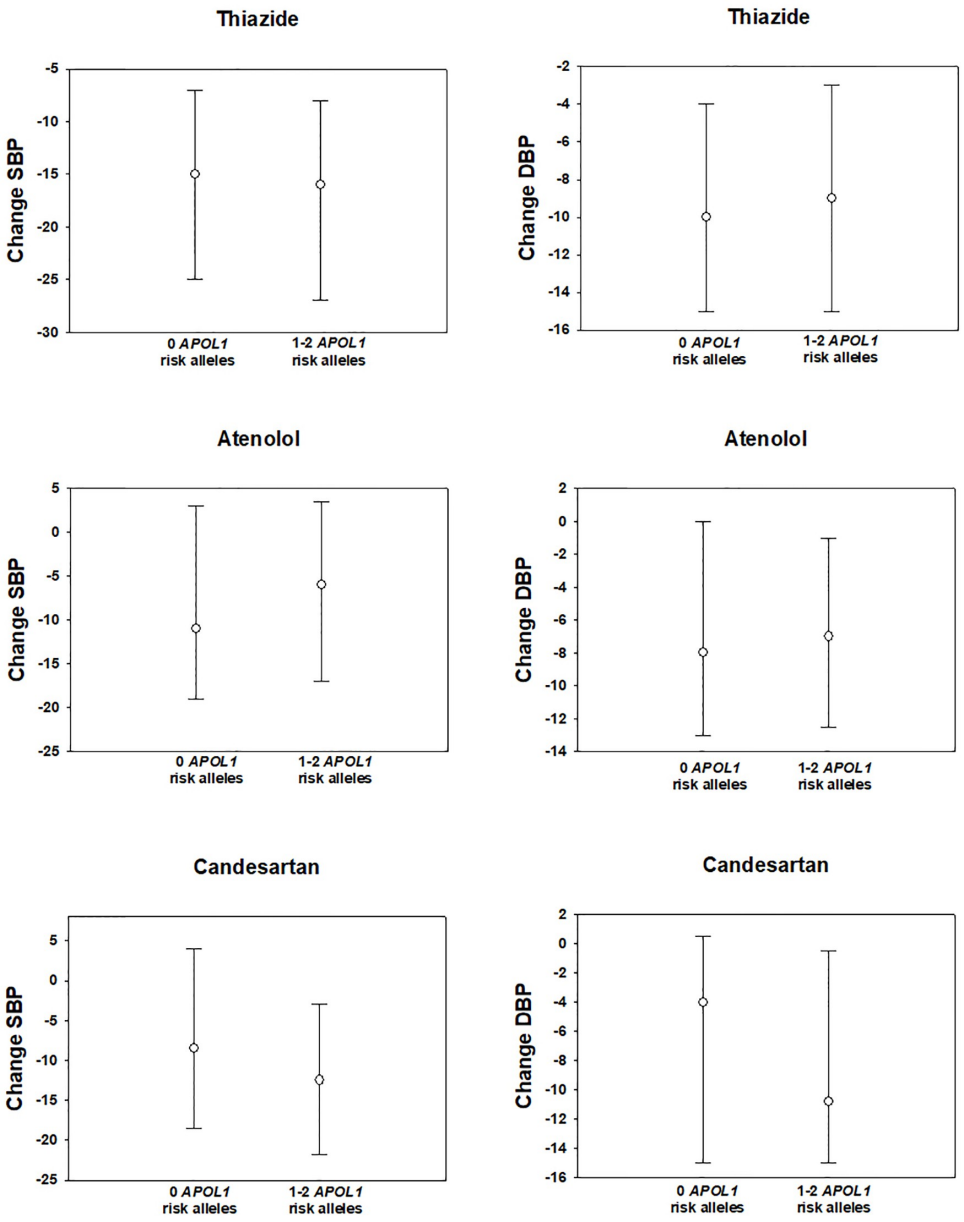
Median and interquartile range for BP response according to *APOL1* genotype. Median SBP and DBP are shown for thiazide, atenolol, and candesartan groups according to 0 versus 1–2 *APOL1* risk alleles.

The magnitude of SBP and DBP decrease with thiazide diuretics (GERA1, PEAR1, and PEAR2), as well as with beta blockers (PEAR1 and PEAR2), did not significantly differ according to the number of *APOL1* risk alleles. This was true of both raw and adjusted BP and was true for BP measured in clinic, at home, or by automated ambulatory BP cuff. This absence of effect for thiazide and beta blocker response was consistent whether *APOL1* risk was analyzed as a having a potential recessive or dominant effect (Table 4 and S3 Table). Response to candesartan in association with *APOL1* genotype is shown in Table 4. Study participants with 1–2 *APOL1* risk alleles demonstrated a greater SBP decrease with candesartan (-12.2 +/- 1.2 vs -7.5 +/- 1.8 mmHg, *p* = 0.03), which reached nominal significance, and a similar trend with a greater DBP response (-8.9 +/- 0.9 vs -6.3 +/- 1.2 mmHg, *p* = 0.08) after adjusting for baseline BP, age, gender, and racial admixture. Fig 1 depicts the median and interquartile range for BP response according to *APOL1* genotype. Participants from the GERA2 cohort in which candesartan was studied did have a significantly higher eGFR when compared to the other three combined cohorts (113.1 +/- 20.1 versus 101.1 +/- 17.9 ml/min, S4 Table) and somewhat lower BP at baseline, perhaps related to the slightly lower age cutoff for GERA1 and GERA2. However, inclusion of eGFR among these factors for BP adjustment did not affect the statistical significance of the results. Similarly, participants with 1–2 *APOL1* risk alleles had a greater decline in albuminuria with candesartan therapy (adjusted values -8.3 +/- 3.1 vs. +3.7 +/- 4.3 mg/day, *p* = 0.02). There were no significant differences between genotype groups in the change in plasma renin activity or serum aldosterone concentration after starting candesartan. Response to candesartan was not significantly different between groups when analyzing *APOL1* genotype according to a recessive or additive risk pattern (S3 and S6 Tables).

**Table 4:** Changes with blood pressure drugs by *APOL1* genotype.

	*APOL1*: 0 risk alleles		*APOL1*: 1–2 risk alleles		P value
	N	Unadjusted mean (SD)	N	Unadjusted Mean (SD)	
Thiazide, clinic SBP change (mmHg)	230	-15.7 (14.2)	340	-17.0 (13.7)	NS
Thiazide, clinic DBP change	230	-9.5 (8.7)	340	-9.2 (8.2)	NS
Atenolol, clinic SBP change	139	-8.9 (15.3)	176	-7.1 (17.5)	NS
Atenolol, clinic DBP change	139	-7.8 (9.3)	176	-7.5 (9.2)	NS
Candesartan, clinic SBP change	65	-6.9 (14.3)	128	-12.4 (14.7)	0.03
Candesartan, clinic DBP change	65	-6.1 (10.7)	128	-9.0 (9.8)	0.08
Candesartan, change in urine albumin (mg/day)	35	-0.1 (23.9)	68	-6.3 (47.2)	0.02
Candesartan, serum K change	64	0.00 (0.36)	127	+0.04 (0.39)	0.21
Candesartan, Aldosterone change	64	-0.89 (4.52)	118	-1.64 (3.49)	0.18
Candesartan, PRA change	64	1.24 (3.70)	127	2.16 (4.46)	0.24

Unadjusted mean and standard deviation (SD) for change in parameters in these three separate trials is shown. P value shown is adjusted for previously identified predictive factors, namely baseline BP, age, gender, PC 1, and PC 2.

**Fig 2:**
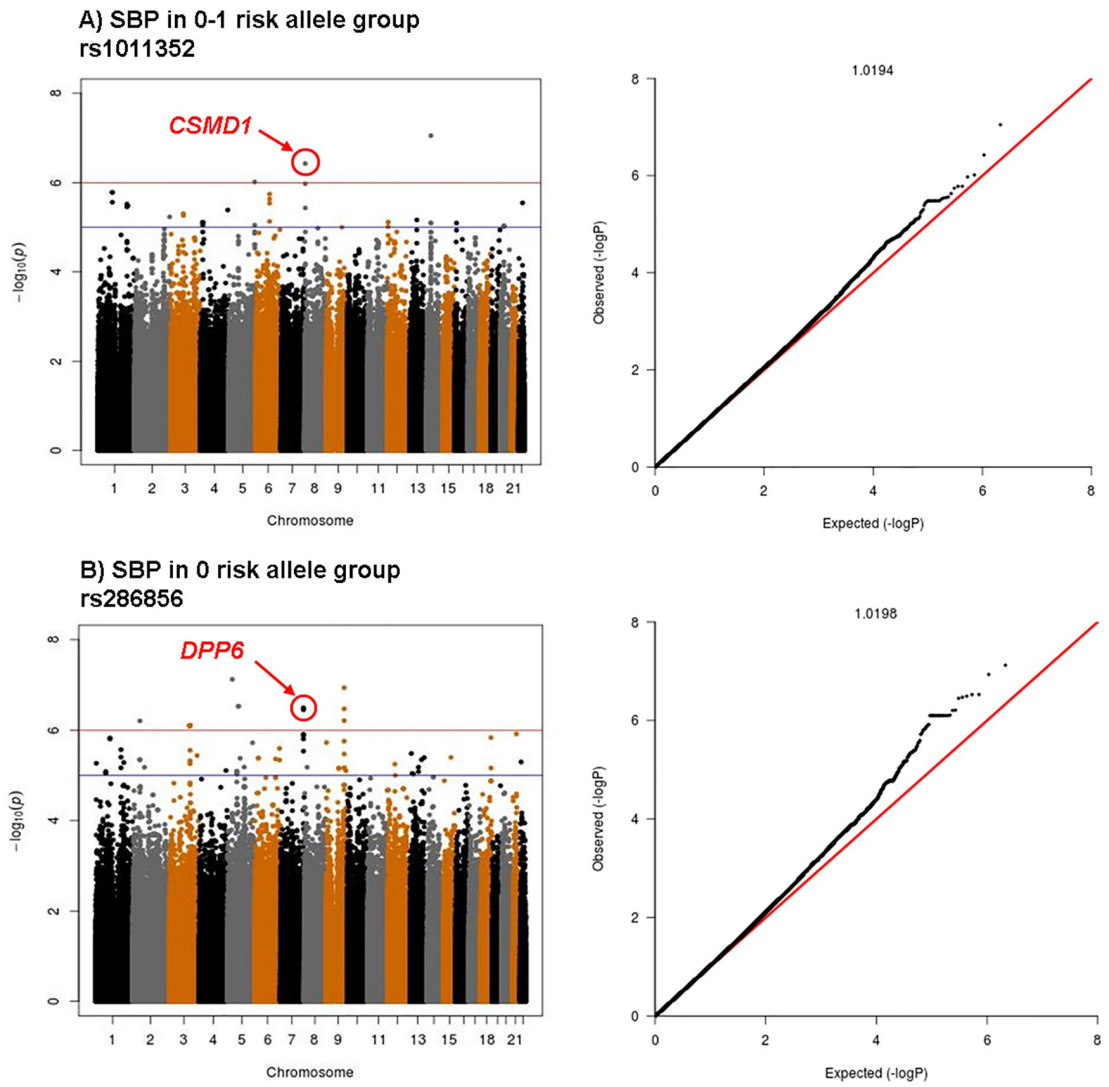
GWAS for SBP response to candesartan. A) Group with 1–2 *APOL1* risk alleles, n = 128. B) Group with 0 *APOL1* risk alleles, n = 65. Manhattan plot and Quantile-Quantile plot with lambda values are shown for each.

Given the differences observed with regard to SBP response to candesartan in GERA2 based on *APOL1* genotype, two GWAS analyses were separately performed in those with 1–2 *APOL1* risk alleles, and separately those with 0 risk alleles, using the magnitude of adjusted SBP response as the outcome of interest. Fig 2A shows the Manhattan and Quantile-Quantile plots for clinic SBP response in the 128 GERA2 patients with one or more *APOL1* risk alleles. Although none achieved a level of Bonferroni corrected genome wide significance (p < 10^−8^), some notable findings were observed. SNP rs10113352, intronic to CUB and Sushi Multiple Domains 1, *CSMD1*, was associated with a greater SBP response to candesartan in individuals with 1–2 *APOL1* risk alleles (*p* = 3.7 x 10^−7^). Fig 2B shows Manhattan and Quantile-Quantile plots for clinic SBP response in the 65 patients with no *APOL1* risk alleles. SNP rs286856, intronic to Dipeptidyl Peptidase Like 6, *DPP6*, was associated with a greater SBP response to candesartan in *APOL1* negative individuals (*p* = 3.2 x 10^−7^). We have highlighted these SNPs because of their statistical significance, as well as factors including their location within the gene and possible biological plausibility. Other SNPs with p < 1.0 x 10^−6^ in this analysis are shown in S5 Table.

## Discussion

Much attention has been recently directed toward understanding the renal consequences of patients of sub-Saharan ancestry carrying allele variants of the *APOL1* gene [28]. *APOL1* is expressed in a variety of tissues throughout the body, including the liver, prostate, placenta and blood vessels [29], however clinical manifestations described to date have been mostly related to the kidney. More recently, a variety of studies have evaluated the role of *APOL1* in cardiovascular disease [5–9]. However, not all individuals with 2 *APOL1* risk alleles will develop kidney disease, and we have relatively little knowledge of the natural history of *APOL1* risk carriers. Some recent work has suggested that a single *APOL1* risk allele may be associated with cardiovascular outcomes [30, 31]. In this study we had the opportunity to evaluate *APOL1* risk allele carriers with mild to moderate essential hypertension, without overt kidney disease, and have gained some new insights as to their clinical characteristics. This is the first study to look at genetic associations of angiotensin receptor blocker response in a primarily AA population.

The prevalence of two *APOL1* risk alleles in our combined cohort (14.8%) was similar to the approximate 13% prevalence previously reported in the US population [28]. The duration of hypertension was greater in individuals with a single *APOL1* risk variant. Baseline BP after four weeks of washout of antihypertensive medications did not differ between groups depending on *APOL1* genotype, regardless of whether it was measured in clinic, at home, by either traditional sphygmomanometer, or at home by automated oscillometric cuff. This suggests that the mechanism through which *APOL1* mutations lead to kidney disease is not due to more prevalent or more severe hypertension. While this study detected no blood pressure differences, only those with mild to moderate hypertension (three antihypertensive medications or less) were eligible for enrollment. It is possible that blood pressure differences do exist between those with and without *APOL1* risk alleles with more severe hypertension, or in those with overt kidney disease. Similar to other studies [32], a significant difference was found in baseline kidney function measured by serum creatinine and eGFR in those with two *APOL1* risk alleles, without significant differences detected in baseline albuminuria. Although much research has focused on the podocyte as a site of APOL1-induced injury, a difference in eGFR occurring without albuminuria suggests the mechanism of renal disease may not limited to the glomerulus or may affect the glomerulus in patterns beyond classic focal segmental glomerulosclerosis [33]. This study provides new insights into possible renal physiologic differences in variant allele carriers individuals before proteinuria or overt kidney failure could confound or distort the picture.

AA patients with essential hypertension have repeatedly been shown to be more salt sensitive and more responsive to diuretics, with less activation of the circulatory renin-angiotensin-aldosterone system, and less sensitivity to ACE inhibition or angiotensin receptor blockade. However, in our analysis of hypertensive AA participants from the GERA2 study, participants with one or more risk alleles for *APOL1* were significantly more responsive to candesartan monotherapy. Importantly, this enhanced BP response was seen without any differences in baseline renin or aldosterone levels (although overall our AA hypertensive patients still had relatively suppressed renin and aldosterone levels compared to their Caucasian counterparts) [16]. However, intra-renal activation of the renin angiotensin system can be present without any alterations in circulatory levels, which only give a relatively crude insight into the level of renin-angiotensin-aldosterone activation at the tissue level. In fact, increased activation of the intrarenal renin-angiotensin system has been described in AA patients [34, 35]. Due to increasingly recognized local renin-angiotensin activation in the kidney, heart, vasculature, and elsewhere [36, 37], it is plausible that angiotensin receptor blockade specifically benefits hypertensive AA patients who carry 1 or more *APOL1* risk alleles. In contrast to candesartan, our analysis found no difference in BP sensitivity to thiazide diuretics or beta blockers in association with the number of *APOL1* risk alleles. The mechanisms that would explain this difference merit further study.

Because of the increased blood pressure response to candesartan in AA participants with 1–2 *APOL1* risk alleles, we performed two separate GWAS analyses of systolic blood pressure response after stratifying for *APOL1* genotype. SNP rs10113352, located within an intron of *CSMD1*, was associated with greater SBP response to candesartan in AA participants with one or more *APOL1* risk alleles. The gene *CSMD1* encodes the protein CUB and Sushi Multiple Domains 1 protein, which is a large transmembrane protein expressed in brain and epithelial tissues, including kidney, which regulates the classical complement system [38]. In previous GWAS analyses, *CSMD1* has been noted to have a possible role in the effect of dietary sodium on blood pressure in a Chinese population, and sensitivity to thiazide diuretics in a Caucasian population [39, 40]. Interestingly, the previous studies found associations with a different SNP, and validation within an Italian population did not find any association with response to valsartan. Additionally, *CSMD1* was found to associate with baseline BP in a Korean cohort [41]. The mechanism by which this gene may affect BP is unknown, but it has also been described to associate with peripheral arterial disease [42]. The fact that our association between SNP rs10113352 and response to candesartan was seen only in patients with one or more *APOL1* risk alleles implies there may be some interaction with *APOL1*. In contrast, those hypertensive AA patients without *APOL1* risk variants had significantly greater SBP response to candesartan in association with rs286856, located within an intron of *DPP6*. DPP6 is inactive as a protease but is a transmembrane protein with several splice variants which associates with voltage gated potassium channels [43, 44]. Variants have been associated with various neurologic abnormalities [45–47], as well as idiopathic ventricular fibrillation [48], and could conceivably play a role in vascular tone and hypertension in AAs. The associations we describe here are preliminary and should be interpreted with caution given the relatively small number of participants, and the fact that that these variants did not achieve genome wide significance. Although there is some biologic rationale to support a potential role for these variants in HTN, these findings will require future validation in other cohorts.

This study is limited by several factors. In recruiting patients without overt kidney disease, but with clearly established essential hypertension, we may be looking at a subset with unique environmental and genetic characteristics, which may not fully apply to the natural history of *APOL1* associated kidney disease in those without HTN, nor to patients with advanced CKD. While this cohort has assembled a significant group of AA subjects, similar to the size of the AASK trial [49], less than half of this group underwent automated blood pressure monitoring, so the power of this study to detect differences in ambulatory blood pressure, or daytime versus nocturnal blood pressure, is less than its power to measure simple office blood pressure. Our findings of different sensitivity to candesartan according to *APOL1* genotype attained a modest degree of statistical significance and should be interpreted with caution given the relatively small number of participants in GERA2. As a *post hoc* analysis of previous trials looking at three different classes of antihypertensives, the nominal significance of this association may be due to a true influence of *APOL1* genotype on the renin angiotensin system, but will clearly require validation in another cohort before this finding can be accepted with confidence. To date no pharmacogenomics trials have examined angiotensin receptor blockers or ACE inhibitors in an AA population; other pharmacogenomic studies of angiotensin receptor blockade have focused on Caucasian participants [19, 50, 51]. Short term trials such as GERA and PEAR are unable to track the longitudinal development of health outcomes or mortality, but the insights into pathophysiology provided helps identify more refined questions.

In summary, this post-hoc analysis provides insights into differences in the characteristics of hypertensive AAs with *APOL1* risk alleles, and is the first to study the effect of *APOL1* genotype on the response to antihypertensive drugs of different classes. The differential sensitivity to angiotensin receptor blockade may also help to unravel conflicting reports about the effect of *APOL1* status on cardiovascular outcomes. More importantly, greater responsiveness to angiotensin receptor blockade could change much of our current practice in how we treat essential hypertension in AA patients without overt kidney disease [52], potentially leading to improved cardiovascular and renal outcomes. Recent work in the AASK cohort found that tight BP control in *APOL1* high risk hypertensive individuals led to a significant improvement in mortality, which was not seen in *APOL1* low risk hypertensive patients [53], demonstrating the importance of optimizing BP control in this group. Nevertheless, AA participants in our analysis still demonstrated the greatest BP response to thiazide diuretics as compared to candesartan or beta blockers, regardless of *APOL1* genotype. Our identification of *CSMD1* and *DPP6* as possible candidate genes which may interact with *APOL1* through podocyte or potassium channel changes with regard to angiotensin receptor blockade response remains to be validated in other studies. As we better understand the mechanisms by which *APOL1* genetic variation associates with renal injury [54, 55], the proteins and pathways identified here may provide further understanding as to how alterations in *APOL1* function result in variable penetrance and phenotype.

## Supporting information

S1 Table Summary of *APOL1* genotyping and imputation methods. (DOCX)

S2 Table Baseline characteristics, recessive model. (DOCX)

S3 Table Changes with blood pressure drugs by *APOL1* genotype, recessive model. (DOCX)

S4 Table Baseline characteristics of GERA2 cohort versus remaining cohorts. (DOCX)

S5 Table Other SNPs associated with blood pressure response, according to *APOL1* genotype. (DOCX)

S6 Table Changes with blood pressure drugs by *APOL1* genotype, additive model. (DOCX)
