# Supplementary material for: Hypertensive APOL1 risk allele carriers demonstrate greater blood pressure reduction with angiotensin receptor blockade compared to low risk carriers
Source: PLoS One. 2019 Sep 18;14(9):e0221957. doi: 10.1371/journal.pone.0221957 (PMC6750571; doi:10.1371/journal.pone.0221957)
Supplement: S1 Table — (DOCX) [file pone.0221957.s001.docx]

| \| **S1 Table. Summary of *APOL1* genotyping and imputation methods.** \| \| \| \| \| \| \| \| \| \| \| --- \| --- \| --- \| --- \| --- \| --- \| --- \| --- \| --- \| --- \| \| **dbSNP ID** \| **CHR** \| **POS** \| **REF** \| **ALT** \| **ALT FREQ** \| **Genotyping Method** \| **SNP Call Rate** \| **Imputation Reference** \| **Rsq** \| \| **PEAR 1** \| \| \| \| \| \| \| \| \| \| \| rs73885319 \| 22 \| 3.7E+07 \| A \| G \| 0.200 \| Illumina HumanExome BeadChip \| 99.7% \| NA \| NA \| \| rs60910145 \| 22 \| 3.7E+07 \| T \| G \| 0.200 \| Illumina HumanExome BeadChip \| 100.0% \| NA \| NA \| \| rs71785313 \| 22 \| 3.7E+07 \| AATAA \| D \| 0.140 \| NA \| 100.0% \| 1000 genome phase 1; Build 37 \| 0.92 \| \| **PEAR 2** \| \| \| \| \| \| \| \| \| \| \| rs73885319 \| 22 \| 3.7E+07 \| A \| G \| 0.195 \| Illumina HumanExome BeadChip \| 99.7% \| NA \| NA \| \| rs60910145 \| 22 \| 3.7E+07 \| T \| G \| 0.196 \| Illumina HumanExome BeadChip \| 100.0% \| NA \| NA \| \| rs71785313 \| 22 \| 3.7E+07 \| AATAA \| D \| 0.177 \| NA \| 100.0% \| 1000 genome phase 1; Build 37 \| 0.93 \| \| **GERA 1** \| \| \| \| \| \| \| \| \| \| \| rs73885319 \| 22 \| 3.7E+07 \| A \| G \| 0.249 \| Illumina HumanExome BeadChip \| 99.7% \| NA \| NA \| \| rs60910145 \| 22 \| 3.7E+07 \| T \| G \| 0.249 \| Illumina HumanExome BeadChip \| 100.0% \| NA \| NA \| \| rs71785313 \| 22 \| 3.7E+07 \| AATAA \| D \| 0.127 \| TaqMan \| 98.7% \| NA \| NA \| \| **GERA 2** \| \| \| \| \| \| \| \| \| \| \| rs73885319 \| 22 \| 3.7E+07 \| A \| G \| 0.258 \| Illumina HumanExome BeadChip \| 99.7% \| NA \| NA \| \| rs60910145 \| 22 \| 3.7E+07 \| T \| G \| 0.258 \| Illumina HumanExome BeadChip \| 100.0% \| NA \| NA \| \| rs71785313 \| 22 \| 3.7E+07 \| AATAA \| D \| 0.169 \| NA \| 100.0% \| 1000 genome phase 1; Build 37 \| 0.95 \| \| Abbreviations: CHR - chromosome, POS - base pair position, REF - reference allele, ALT - alternate allele (D=deletion), ALT FREQ - alternate allele frequency, QC - quality control, SNP - single nucleotide polymorphism, Rsq - R squared, NA - not applicable \| \| \| \| \| \| \| \| \| \| |
| --- | --- | --- | --- | --- | --- | --- | --- | --- | --- | --- | --- | --- | --- | --- | --- | --- | --- | --- | --- | --- | --- | --- | --- | --- | --- | --- | --- | --- | --- | --- | --- | --- | --- | --- | --- | --- | --- | --- | --- | --- | --- | --- | --- | --- | --- | --- | --- | --- | --- | --- | --- | --- | --- | --- | --- | --- | --- | --- | --- | --- | --- | --- | --- | --- | --- | --- | --- | --- | --- | --- | --- | --- | --- | --- | --- | --- | --- | --- | --- | --- | --- | --- | --- | --- | --- | --- | --- | --- | --- | --- | --- | --- | --- | --- | --- | --- | --- | --- | --- | --- | --- | --- | --- | --- | --- | --- | --- | --- | --- | --- | --- | --- | --- | --- | --- | --- | --- | --- | --- | --- | --- | --- | --- | --- | --- | --- | --- | --- | --- | --- | --- | --- | --- | --- | --- | --- | --- | --- | --- | --- | --- | --- | --- | --- | --- | --- | --- | --- | --- | --- | --- | --- | --- | --- | --- | --- | --- | --- | --- | --- | --- | --- | --- | --- | --- | --- | --- | --- | --- | --- | --- | --- | --- | --- | --- | --- | --- | --- | --- | --- | --- | --- | --- | --- | --- | --- | --- | --- | --- | --- |
