# Supplementary material for: Hypertensive APOL1 risk allele carriers demonstrate greater blood pressure reduction with angiotensin receptor blockade compared to low risk carriers
Source: PLoS One. 2019 Sep 18;14(9):e0221957. doi: 10.1371/journal.pone.0221957 (PMC6750571; doi:10.1371/journal.pone.0221957)
Supplement: S2 Table — (DOCX) [file pone.0221957.s002.docx]

**S2 Table. Baseline characteristics, recessive model.**

|  | **APOL1:**  **0-1 risk alleles**  **819 total** |  | **APOL1:**  **2 risk alleles**  **142 total** |  |  |
| --- | --- | --- | --- | --- | --- |
|  | **N** | **Mean (SD)** | **N** | **Mean (SD)** | **P value** |
| Gender (% female) |  | 56.7 |  | 52.8 | NS |
| Age | 819 | 48.3 (7.77) | 142 | 48.3 (7.55) | NS |
| Waist/Hip | 654 | 0.87 (0.08) | 110 | 0.89 (0.09) | NS |
| BMI | 819 | 31.3 (5.81) | 142 | 30.7 (5.60) | NS |
| Hypertension duration | 754 | 7.4 (7.36) | 131 | 8.0 (8.07) | NS |
| Hypertension onset age | 494 | 40.3 (9.20) | 73 | 38.8 (9.74) | 0.19 |
| Albumin (g/dl) | 615 | 3.96 (0.40) | 103 | 3.93 (0.30) | NS |
| Hemoglobin (g/dl) | 662 | 14.0 (7.98) | 110 | 13.6 (1.44) | NS |
|  |  |  |  |  |  |
| Clinic SBP, baseline | 819 | 150.0 (13.6) | 142 | 149.7 (13.5) | NS |
| Clinic DBP, baseline | 819 | 97.7 (6.0) | 142 | 97.6 (5.8) | NS |
| Home SBP, baseline | 420 | 146.8 (11.0) | 68 | 145.9 (11.5) | NS |
| Home DBP, baseline | 420 | 94.8 (6.4) | 68 | 95.5 (6.5) | NS |
| Ambulatory SBP, all | 317 | 140.1 (12.4) | 48 | 140.1 (10.0) | NS |
| Ambulatory DBP, all | 317 | 88.7 (8.4) | 48 | 89.5 (7.0) | NS |
| Ambulatory SBP, day | 317 | 142.8 (12.7) | 48 | 142.3 (10.2) | NS |
| Ambulatory DBP, day | 317 | 91.3 (8.6) | 48 | 91.7 (7.1) | NS |
| Ambulatory SBP, night | 314 | 133.1 (14.0) | 47 | 134.8 (12.0) | NS |
| Ambulatory DBP, night | 314 | 82.1 (10.1) | 47 | 83.9 (9.1) | NS |
|  |  |  |  |  |  |
| Urine Na, baseline (meq/24 h) | 651 | 146.5 (72.0) | 109 | 137.4 (61.5) | NS |
| Serum Na, baseline | 817 | 139.5 (4.6) | 141 | 140.1 (3.7) | NS |
| Serum K, baseline | 815 | 3.98 (0.42) | 142 | 4.03 (0.35) | NS |
| Serum creatinine, baseline | 817 | 0.87 (0.21) | 110 | 0.93 (0.24) | 0.0055 |
| Serum aldosterone | 778 | 7.57 (5.75) | 136 | 7.90 (5.20) | NS |
| Serum renin | 806 | 0.66 (1.12) | 139 | 0.61 (0.59) | NS |
| Urine alb/creat (mcg/mg) | 568 | 23 (92) | 65 | 20 (31) | NS |
| eGFR (ml/min) | 817 | 104.4 (18.7) | 142 | 98.7 (19.6) | 0.0008 |
