# Supplementary material for: Hypertensive APOL1 risk allele carriers demonstrate greater blood pressure reduction with angiotensin receptor blockade compared to low risk carriers
Source: PLoS One. 2019 Sep 18;14(9):e0221957. doi: 10.1371/journal.pone.0221957 (PMC6750571; doi:10.1371/journal.pone.0221957)
Supplement: S3 Table — (DOCX) [file pone.0221957.s003.docx]

**S3 Table. Changes with blood pressure drugs by *APOL1* genotype, recessive model.**

|  | ***APOL1*:**  **0-1 risk alleles** |  |  | ***APOL1*:**  **2 risk alleles** |  |  |
| --- | --- | --- | --- | --- | --- | --- |
|  | **N** | **Adjusted**  **mean (SEM)** |  | **N** | **Adjusted**  **Mean (SEM)** | **P value** |
| Thiazide, clinic SBP change (mmHg) | 485 | -16.0 (0.6) |  | 85 | -16.5 (1.3) | NS |
| Thiazide, clinic DBP change | 485 | -9.1 (0.4) |  | 85 | -9.2(0.9) | NS |
|  |  |  |  |  |  |  |
| Atenolol, clinic SBP change | 270 | -8.2 (0.9) |  | 45 | -6.4(2.3) | NS |
| Atenolol, clinic DBP change | 270 | -7.8 (0.5) |  | 45 | -6.1 (1.3) | NS |
|  |  |  |  |  |  |  |
| Candesartan, clinic SBP change | 156 | -10.4 (1.1) |  | 37 | -11.2 (2.4) | NS |
| Candesartan, clinic DBP change | 156 | -7.8 (0.8) |  | 37 | -9.0 (1.7) | NS |
|  |  |  |  |  |  |  |

Patients are taken from the four studies described in Tables 1, but grouped differently by genotype for 0-1 versus 2 risk alleles. Group means, SEM, and P values are adjusted for previously identified predictive factors, namely baseline BP, age, gender, PC 1, and PC 2.
