# Supplementary material for: Hypertensive APOL1 risk allele carriers demonstrate greater blood pressure reduction with angiotensin receptor blockade compared to low risk carriers
Source: PLoS One. 2019 Sep 18;14(9):e0221957. doi: 10.1371/journal.pone.0221957 (PMC6750571; doi:10.1371/journal.pone.0221957)
Supplement: S4 Table — (DOCX) [file pone.0221957.s004.docx]

**S4 Table. Baseline characteristics of GERA2 cohort versus remaining cohorts.**

|  | PEAR1, PEAR2, GERA1  (n = 768) | GERA2  (n = 193) | P value |
| --- | --- | --- | --- |
|  |  |  |  |
| Gender (% female) | 57.6 | 50.3 | NS |
| Age | 48.2 (8.00) | 48.7 (6.60) | NS |
| Waist/Hip | 0.87 (0.08) | 0.90 (0.08) | < 0.0001 |
| BMI | 31.4 (6.00) | 30.4 (4.73) | 0.0092 |
| Hypertension Duration | 7.4 (7.18) | 7.6 (8.46) | NS |
| Hypertension Age | 40.1 (9.28) | 41.1 (9.80) | NS |
| Albumin (g/dl) | 3.98 (0.34) | 3.90 (0.39) | 0.015 |
| Hemoglobin (g/dl) | 13.49 (1.54) | 13.86 (2.42) | 0.044 |
|  |  |  |  |
| Clinic SBP, baseline | 150.7 (13.8) | 147.2 (12.4) | 0.0012 |
| Clinic DBP, baseline | 98.2 (6.0) | 95.7 (5.0) | < 0.0001 |
|  |  |  |  |
| Urine Na, baseline (meq/24 h) | 145.5 (67.5) | 144.5 (79.5) | NS |
| Serum Na, baseline | 139.4 (2.8) | 140.2 (2.89) | NS |
| Serum K, baseline | 3.98 (0.42) | 4.03 (0.37) | NS |
| Serum creatinine, baseline | 0.90 (0.21) | 0.80 (0.23) | < 0.0001 |
| Serum aldosterone | 7.59 (5.92) | 7.72 (4.49) | NS |
| Serum renin | 0.65 (0.65) | 0.66 (2.02) | NS |
| Urine alb/creat (mcg/mg) | 23 (78) | 24 (120) | NS |
| eGFR (ml/min) | 101.1 (17.9) | 113.3 (20.1) | < 0.0001 |
|  |  |  |  |
