# Supplementary material for: Hypertensive APOL1 risk allele carriers demonstrate greater blood pressure reduction with angiotensin receptor blockade compared to low risk carriers
Source: PLoS One. 2019 Sep 18;14(9):e0221957. doi: 10.1371/journal.pone.0221957 (PMC6750571; doi:10.1371/journal.pone.0221957)
Supplement: S5 Table — (DOCX) [file pone.0221957.s005.docx]

**S5 Table. Other SNPs associated with blood pressure response, according to *APOL1* genotype.**

| **SNP** | **gene** | **chrom.** | **beta** | **SD** | **p value** | **location relative  to gene** | **function** | **possible associations** |
| --- | --- | --- | --- | --- | --- | --- | --- | --- |
|  |  |  |  |  |  |  |  |  |
| **SBP response  1-2 risk alleles** |  |  |  |  |  |  |  |  |
| rs4900726 | MDGA2, MAM domain containing  glycosylphosphatidylinositol anchor 2 | 14 | -9.88 | 1.85 | 8.91E-08 | intronic | neuron cell adhesion, carnitine | anxiety disorders |
| rs10113352 | CSMD1, CUB and sushi multiple domains 1 | 8 | -9.69 | 1.9 | 3.74E-07 | intronic | complement regulation | see text; HTN |
| rs17069831 | CSMD1, CUB and sushi multiple domains 1 | 8 | -9.92 | 2.03 | 1.06E-06 | intronic | complement regulation | see text; HTN |
| rs2042158 | MIR4634 | 5 | -9.05 | 1.84 | 9.65E-07 | intronic | possible role in ERBB2 /HER2 gene (breast) |  |
| rs11165656 | signal recognition particle RNA | 1 | 16.01 | 3.36 | 1.65E-06 | 5' 88kB | signal peptide processing |  |
| rs12030482 | signal recognition particle RNA | 1 | 16.09 | 3.36 | 1.66E-06 | 5' 88kB | signal peptide processing |  |
| rs2800426 | signal recognition particle RNA | 1 | 15.91 | 3.39 | 2.75E-06 | 5' 69 kB | signal peptide processing |  |
| rs1324115 | EPH A7 receptor | 6 | -8.46 | 1.77 | 1.81E-06 | 3' 159 kb | tyrosine kinase |  |
| rs1570632 | EPH A7 receptor | 6 | -8.34 | 1.77 | 2.33E-06 | 3' 159 kb | tyrosine kinase |  |
| rs5751125 | CSDC2, cold shock domain containing 2 | 22 | 10.82 | 2.31 | 2.84E-06 | 5' 3.5 kB | RNA binding protein, in brain |  |
|  |  |  |  |  |  |  |  |  |
|  |  |  |  |  |  |  |  |  |
| **DBP response  1-2 risk alleles** |  |  |  |  |  |  |  |  |
| rs7704226 | NREP, neuronal regulation related protein | 5 | 5.67 | 1.12 | 4.32E-07 | intronic | wound healing; renal fibrosis; | schizophrenia |
| rs6898959 | NREP, neuronal regulation related protein | 5 | 5.67 | 1.12 | 4.38E-07 | intronic | wound healing; renal fibrosis | schizophrenia |
| rs6898801 | NREP, neuronal regulation related protein | 5 | 5.66 | 1.12 | 4.59E-07 | intronic | wound healing; renal fibrosis | schizophrenia |
| rs26557 | NREP, neuronal regulation related protein | 5 | 5.59 | 1.11 | 4.91E-07 | intronic | wound healing; renal fibrosis | schizophrenia |
| rs6741429 | PLCL1, phospholipase C-like 1 | 2 | 5.97 | 1.21 | 8.29E-07 | 3', 530kb | phospholipase activity |  |
| rs1455334 | PLCL1, phospholipase C-like 1 | 2 | 5.97 | 1.21 | 8.33E-07 | 3', 530kb | phospholipase activity |  |
| rs1455335 | PLCL1, phospholipase C-like 1 | 2 | 5.97 | 1.21 | 8.45E-07 | 3', 530kb | phospholipase activity |  |
| rs12693858 | PLCL1, phospholipase C-like 1 | 2 | 5.97 | 1.21 | 8.51E-07 | 3', 530kb | phospholipase activity |  |
| rs12116508 | DENND1B, DENN domain containing 1B | 1 | 10.03 | 2.05 | 1.05E-06 | intronic | clathrin process; IBD, asthma |  |
| rs4915557 | DENND1B, DENN domain containing 1B | 1 | 10.03 | 2.06 | 1.05E-06 | intronic | clathrin process; IBD, asthma |  |
|  |  |  |  |  |  |  |  |  |
|  |  |  |  |  |  |  |  |  |
| **SBP response  0 risk alleles** |  |  |  |  |  |  |  |  |
| rs4391181 | CDH6, cadherin 6 | 5 | -19.52 | 3.63 | 7.54E-08 | 5', 1000 kb | kidney development |  |
| rs1911936 | CARTPT, CART prepropeptide | 5 | 18.57 | 3.62 | 2.98E-07 | 3', 124 kb | energy and reward systems | role in cocaine related HTN |
| rs7709962 | CARTPT, CART prepropeptide | 5 | 18.57 | 3.62 | 2.97E-07 | 3', 124 kb | energy and reward systems | role in cocaine related HTN |
| rs1318839 | TLR4, Toll receptor 4 | 9 | 12.2 | 2.3 | 1.16E-07 | 3', 533kb | innate immune signaling | multiple immune related |
| rs10818132 | TLR4, Toll receptor 4 | 9 | 11.82 | 2.32 | 3.38E-07 | 3', 578 kb | innate immune signaling | multiple immune related |
| rs10818136 | TLR4, Toll receptor 4 | 9 | 11.7 | 2.35 | 6.16E-07 | 3', 596 kb | innate immune signaling | multiple immune related |
| rs286856 | DPP6, dipeptydyl peptidase 6 | 7 | -13.69 | 2.68 | 3.21E-07 | intronic | see text |  |
| rs286835 | DPP6, dipeptydyl peptidase 6 | 7 | -13.6 | 2.67 | 3.54E-07 | intronic | see text |  |
| rs11125096 | noncoding RNA | 2 | -9.52 | 1.91 | 6.23E-07 | intronic |  |  |
| rs13097302 | SOX14, sex-determining region Y box 14 | 3 | -14.14 | 2.86 | 7.78E-07 | 5', 360 kb | transcription factor, various congenital  malformations |  |
|  |  |  |  |  |  |  |  |  |
| **DBP response  0 risk alleles** |  |  |  |  |  |  |  |  |
| rs4794488 | SKAP1, src kinase associated phosphoprotein 1 | 17 | 14.24 | 2.61 | 5.12E-08 | intronic | T cell adaptor protein |  |
| rs1018335 | NELL1, NEL Like-1 | 11 | -9.37 | 1.72 | 5.34E-08 | intronic | possible role in cell growth, differentiation | role in metabolic  complications to thiazides |
| rs7326276 | DOCK9, dedicator of cytokinesis 9 | 13 | 8.51 | 1.61 | 1.34E-07 | intronic | cell signaling |  |
| rs13435803 | LPHN3, Iatrophilin 3 | 4 | -10.93 | 2.08 | 1.49E-07 | 3', 407 kb | cell signaling, cell-cell adhesion | Role in ADHD |
| rs10178845 | LINC00299, long noncoding RNA299 | 2 | -9.96 | 1.93 | 2.34E-07 | intronic |  | Role in allergy |
| rs11656941 | SKAP1, src kinase associated phosphoprotein 1 | 17 | -12.87 | 2.53 | 3.80E-07 | intronic | T cell adaptor protein |  |
| rs11864983 | PLCG2, phospholipase C gamma-2 | 16 | 14.48 | 2.87 | 4.52E-07 | intronic | phospholipase | autoimmune findings |
| rs4355854 | MIR4289, microRNA 4289 | 9 | 9.11 | 1.81 | 5.01E-07 | 5', 52 kb |  |  |
| rs698023 | DACT1, dapper antagonist of beta catenin, homolog 1 | 14 | 10.44 | 2.09 | 6.37E-07 | 3', 19 kb | developmental signaling  including Wnt |  |
| rs4391181 | CDH6, cadherin 6 | 5 | -12.93 | 2.6 | 6.82E-07 | 5' 1065 kb | kidney development |  |
