# Supplementary material for: Hypertensive APOL1 risk allele carriers demonstrate greater blood pressure reduction with angiotensin receptor blockade compared to low risk carriers
Source: PLoS One. 2019 Sep 18;14(9):e0221957. doi: 10.1371/journal.pone.0221957 (PMC6750571; doi:10.1371/journal.pone.0221957)
Supplement: S6 Table — (DOCX) [file pone.0221957.s006.docx]

**S6 Table. Changes with blood pressure drugs by *APOL1* genotype, additive model.**

|  | ***APOL1*:**  **0 risk alleles** |  |  | ***APOL1*:**  **1 risk alleles** |  |  | ***APOL1*:**  **2 risk alleles** |  |  |
| --- | --- | --- | --- | --- | --- | --- | --- | --- | --- |
|  | **N** | **Unadjusted**  **mean (SD)** |  | **N** | **Unadjusted**  **Mean (SD)** |  | **N** | **Unadjusted**  **Mean (SD)** | **P value** |
| Thiazide, clinic SBP change (mmHg) | 230 | -15.7 (14.2) |  | 255 | -17.3 (13.8) |  | 85 | -16.0 (13.4) | NS |
| Thiazide, clinic DBP change | 230 | -9.5 (8.7) |  | 255 | -9.1(8.2) |  | 85 | -9.3 (8.5) | NS |
|  |  |  |  |  |  |  |  |  |  |
| Atenolol, clinic SBP change | 139 | -8.9 (15.3) |  | 131 | -7.9 (18.6) |  | 45 | -4.7 (13.8) | NS |
| Atenolol, clinic DBP change | 139 | -7.8 (9.3) |  | 131 | -7.9 (9.6) |  | 45 | -6.1 (7.8) | NS |
|  |  |  |  |  |  |  |  |  |  |
| Candesartan, clinic SBP change | 65 | -6.9 (14.3) |  | 91 | -12.5 (15.3) |  | 37 | -12.1 (13.2) | NS |
| Candesartan, clinic DBP change | 65 | -6.1 (10.7) |  | 91 | -9.1 (9.6) |  | 37 | -8.7 (10.2) | NS |

Patients are taken from the four studies described in Tables 1, but grouped differently by genotype for 0, 1, and 2 risk alleles. Group means and standard deviation are given. P values are adjusted for previously identified predictive factors, namely baseline BP, age, gender, PC 1, and PC 2 did not reach statistical significance.
